# Hybrid Origin of ×*Leymotrigia bergrothii* (Poaceae) as Revealed by Analysis of the Internal Transcribed Spacer ITS1 and *trn*L Sequences

**DOI:** 10.3390/ijms252211966

**Published:** 2024-11-07

**Authors:** Elizaveta O. Punina, Alexander A. Gnutikov, Nikolai N. Nosov, Victoria S. Shneyer, Alexander V. Rodionov

**Affiliations:** 1 Laboratory of Biosystematics and Cytology, Komarov Botanical Institute of the Russian Academy of Sciences, 197022 St. Petersburg, Russia; a.gnutikov@vir.nw.ru (A.A.G.); nnosov@binran.ru (N.N.N.); shneyer@binran.ru (V.S.S.); avrodionov@binran.ru (A.V.R.); 2 Department of Genetic Resources of Oat, Barley, Rye, Federal Research Center N. I. Vavilov All-Russian Institute of Plant Genetic Resources (VIR), 190000 St. Petersburg, Russia

**Keywords:** *Elymus*, *Elytrigia*, hybridization, *Leymus*, molecular phylogeny, Triticeae

## Abstract

×*Leymotrigia bergrothii* is a presumed hybrid of *Leymus arenarius* and *Elytrigia repens*. This article investigates the hybrid origin and genome composition of this species. These plants are sterile, do not undergo pollination, and do not produce seeds; occasionally, underdeveloped stamens containing abortive pollen grains form in individual spikelets. The karyotype analysis of root meristem cells revealed a diploid chromosome number of 49 in ×*L. bergrothii*, reported here for the first time. Subsequently, we examined the intragenomic polymorphism of the transcribed spacer ITS1 in several species of *Elytrigia*, *Elymus*, *Leymus*, *Hordeum*, and *Psathyrostachys*, and compared the ribotype patterns of these species with those of ×*L. bergrothii.* It is shown that the St-ribotype variants found in *Elytrigia repens* and *Elytrigia pseudocaesia*, as well as the ribotypes of the La family, which dominate in the genome of *Leymus arenarius*, correspond to major ribotypes in ×*L. bergrothii*. The ribotypes of the St and La families are present in the nuclear genome of ×*L. bergrothii* in almost equal proportions. A comparison of intron and exon sequences of the *trn*L gene in the chloroplast DNA of *L*ey*mus arenarius*, *Elytrigia repens*, and ×*L. bergrothii* showed that this region in ×*L. bergrothii* is identical or very close to that of *Elytrigia repens*, suggesting that *Elytrigia repens* was the cytoplasmic donor to ×*L. bergrothii.* Thus, our study confirms the hypothesis that this species represents a sterile first-generation hybrid of *Leymus arenarius* and *Elytrigia repens*, reproducing vegetatively.

## 1. Introduction

Within the Poaceae family, several genera and species are believed to have arisen through direct hybridization between two species [1]. These plants are typically classified into specific taxonomic units known as nothogenera and nothospecies. These cases are of general biological interest as they clearly demonstrate the role of interspecies hybridization in plant speciation and evolution. One such nothospecies was described by the eminent Finnish botanist Harald Lindberg as *Tritordeum bergrothii* H. Lindb., presumed to be a hybrid of *Triticum repens* L. and *Hordeum arenarium* (L.) Aschers., based on plants collected by Ivar Ossian Bergroth on the island of Russky Kuzov near the Karelian coast of Onega Bay in the White Sea (64°55′53″ N 35°08′59″ E) and by Aimo Kaarlo Kajander on the eastern shore of Onega Bay near the village of Pokrovskoye (64°01′01″ N 38°05′50″ E, now Onezhsky District, Arkhangelsk Oblast, Russia) in 1896 and 1899 [2]. Plants from the Kajander collection were noted to be morphologically closer to *Hordeum arenarium* f. *subarenarium* (now *Leymus arenarius*), while those from Russky Kuzov Island were more similar to *Triticum repens* f. *subrepens* (now *Elymus repens* according to POWO or *Elytrigia repens*) (Lindberg, 1905–1906). Subsequently, Nikolai Tzvelev classified these plants under the genus ×*Leymotrigia* Tzvelev as the species ×*L. bergrothii* (H. Lindb.) Tzvelev [3]. According to the current taxonomic understanding, it is ×*Leymotrigia bergrothii* (H. Lindb.) Tzvelev (syn. ×*Elyleymus bergrothii* (H. Lindb.) Conert), a hybrid of *Elytrigia repens* (L.) Nevski (syn. *Elymus repens* (L.) Gould), and *Leymus arenarius* Hochst [1,4,5].

The putative parental species have rather wide ranges; thus, *E. repens* can be considered a practically pan-Eurasian species with a probable introduction to North America, and the range of *L. arenarius* covers most of Europe and was introduced to North America [1]. It appears that ×*L. bergrothii* is rarely encountered on the islands of the White Sea and the western coast of Onega Bay [6,7,8,9]. However, the species is quite abundant on the sandy beaches of the eastern coast of Onega Bay [10]. The report that the species also occurs on the sandy shores of Vossinoinsaari Island in Lake Ladoga [7,11] seems doubtful and has not yet been confirmed by herbarium material [12].

Until recently, the hybrid origin of ×*L. bergrothii* could only be inferred due to the unusual combination of morphological traits that are intermediate between those of the parental species [13,14]. However, diagnostic traits determining taxonomic affiliation are sometimes polygenically coded, and in other cases, monogenically. This can differentially influence the mosaic of morphological traits in hybrid offspring, complicating the accurate identification of the parental species [15,16]. Finally, parallels in hereditary variability according to Vavilov’s law of homologous series in variability [17,18] can create the illusion of hybrid origin where it does not exist.

One of the modern approaches to studying the hybrid origin of plants involves examining intragenomic polymorphism of multiple repeated genes, such as 35S rRNA genes [19,20,21,22]. Any plant’s nuclear genome contains several thousand tandemly arranged 35S rRNA genes [23]. Within each 35S rRNA locus, sequences encoding 18S, 5.8S, and 28S rRNA are separated by internal transcribed spacers ITS1 and ITS2, whose variability level allows for their use as DNA identifiers (DNA barcodes) for species [24,25]. To verify the hybrid origin, we investigated the intragenomic polymorphism of the ITS1 spacer of the 35S rRNA genes of ×*L. bergrothii* collected on the sandy shore of the eastern coast of Onega Bay, as well as their presumed parents. Since it is known that the genera *Elytrigia* (*Elymus*) and *Leymus* are hybridogenic allopolyploids, their composite genomes include two to three of several basic subgenomes defined for different representatives of the tribe Hordeeae [26,27], so we also included some species of other genera of this tribe in the comparative analysis. We chose the ITS1 35S rDNA fragment as a marker because homogenization in ITS1 is usually lower than in ITS2 [28,29].

## 2. Results

**Figure 1:**
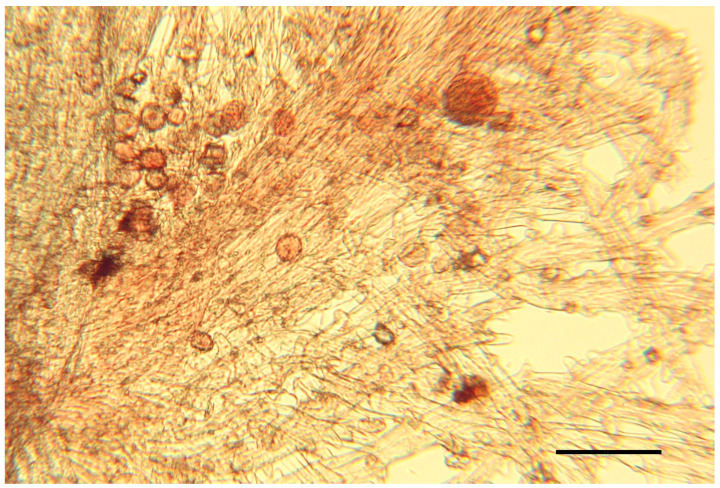
Deformed and uncolored pollen grains in the anthers of ×*Leymotrigia bergrothii.* Acetic-orcein staining. Bar = 100 μm.

The fertility analysis has shown that in all cases we observed unstained pollen grains of ×*Leymotrigia. bergrothii* without cytoplasmic content or lightly stained and deformed ones (Figure 1).

**Figure 2:**
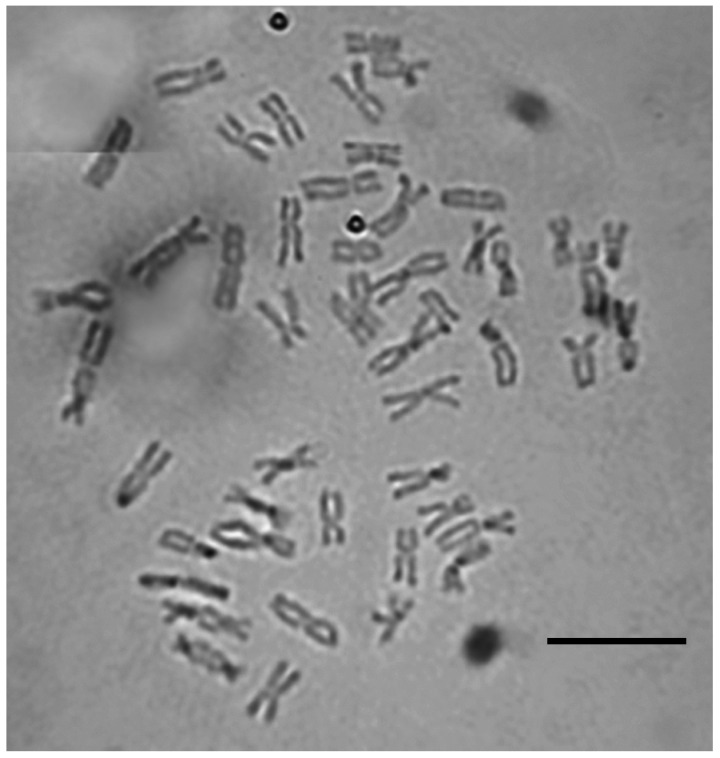
Karyotype of ×*Leymotrigia bergrothii,* 2n = 49, root meristem cell, acetic-orcein staining. Bar = 10 μm.

The karyotype study showed that the collected plants of ×*L. bergrothii* have a diploid set of 2n = 49 chromosomes (Figure 2).

**Figure 3:**
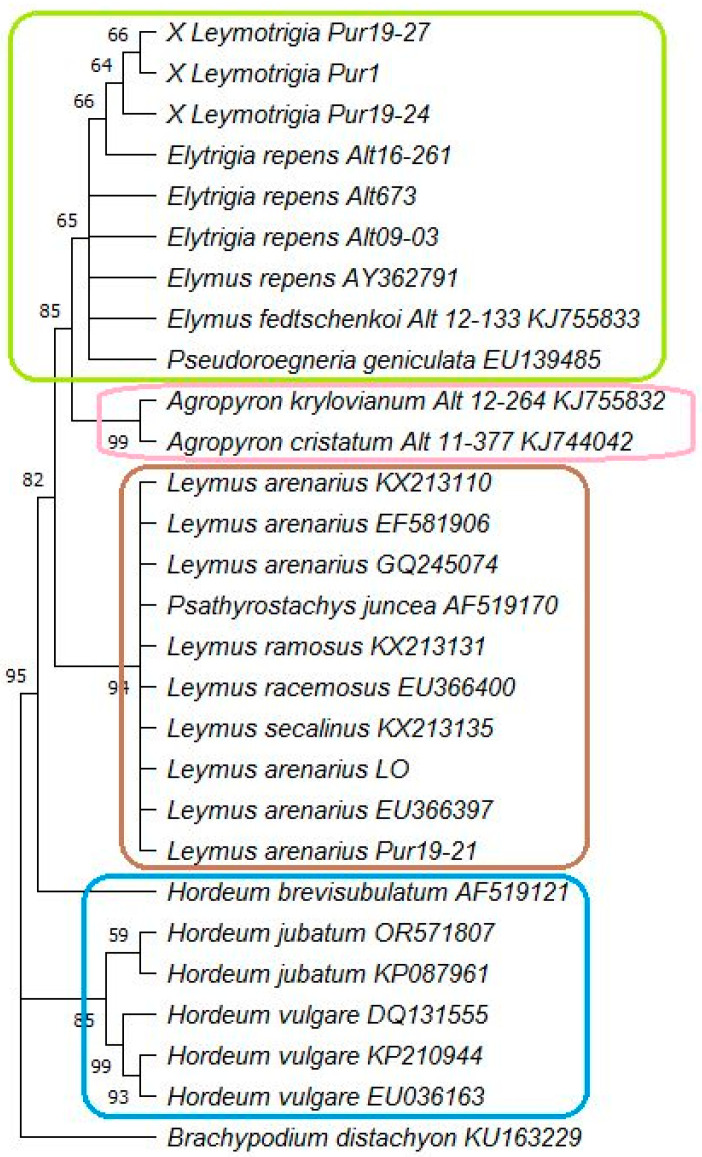
Phylogenetic tree of chloroplast gene *trnL* sequences based on the Minimal Evolution method and Maximum Composite Likelihood model, using 1000 replicates in the Bootstrap test of phylogeny and the GTR + G substitution model. Bootstrap values are indicated above and below branches. Green color box indicates common origin of ×*Leymotrigia bergrothii, Elytrigia, Elymus*, and *Pseudoroegneria* chloroplast sequences. Pink, brownish, and blue colour boxes indicate common origin of *Agropyron*, *Leymus*/*Psathyrostachys*, and *Hordeum* respectively.

The topology of the phylogenetic tree of chloroplast gene *trn*L sequences based on the Minimal Evolution method (Figure 3) was very similar to the topology of that based on the Bayesian method. The sequences of *Leymus arenarius, Elytrigia repens,* and ×*L. bergrothii* are rather similar (Table S1). This region of ×*Leymotrigia* is mostly similar to that of *Elytrigia repens* Alt16-261, collected in the Altai Mountains, differing only by a single nucleotide deletion from the sequences of *Elytrigia repens* Alt673 and Alt09-03 vouchers (Figure 1, Table S1). The *trnL* gene sequences of *Leymus arenarius* differed significantly by seven SNPs and a 5-nucleotide deletion (Table S1). The reference sequence taken from the *Leymus arenarius* GQ245074 sample from the Barents Sea coast, Wrangel Peninsula, Norway [30], is identical to the *trnL* gene sequence of *L. arenarius* collected on the eastern coast of Onega Bay (Pur 19–21) and in Leningrad Oblast (LO), northwest Russia.

We then studied the intragenomic polymorphism of the internal transcribed spacer ITS1 in ×*L. bergrothii* and some related grasses using NGS sequencing on the Illumina platform. The ZOTUs (ribotypes) found in the studied samples are presented in Table S2 (Supplementary Materials). For this study, we used the ITS1 sequence of *Agropyron cristatum* Alt 11-377, GenBank accession number KJ561241, as the reference sequence. The length of the aligned ITS1 sequence from the CGTGACCC motif to the TTTAATC motif was 222 bp.

**Figure 4:**
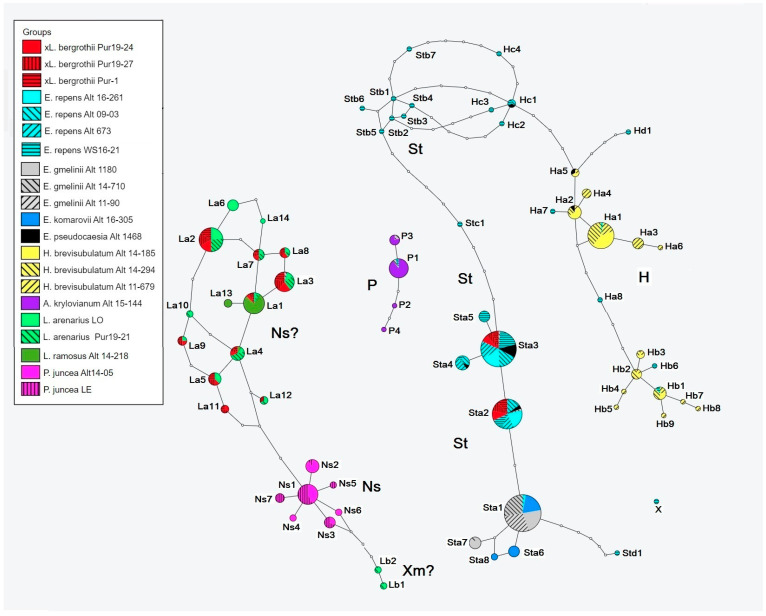
The grass ribotype networks showing the level of intragenome and interspecific variation based on the ITS1 sequences. The network was made by the probabilistic method of Statistical Parsimony [31] by using the TCS 1.21 software [32] and the tcsBU program network visualization [33]. The filled circles represent ZOTUs (ribotypes). Colors represent different species and the sizes of wedges and circles are proportional to the relative abundance of a variant in a specimen.

The ribotypes of the studied species are polymorphic and can be divided into several ribotype families, denoted in the Figure as P, H, St, Ns, Xm, La, and Lb (Figure 4). We used the commonly accepted designations of ribotypes characterizing the individual subgenomes of Hordeeae [26,27] as *Agropyron* (P), *Hordeum*/*Critesion* (H), *Pseudoroegneria* (St), *Psathyrostachys* (Ns), and *Leymus* (Ns, Xm). We designated the new ribotypes of *Leymus* that we identified as La and Lb.

**Figure 5:**
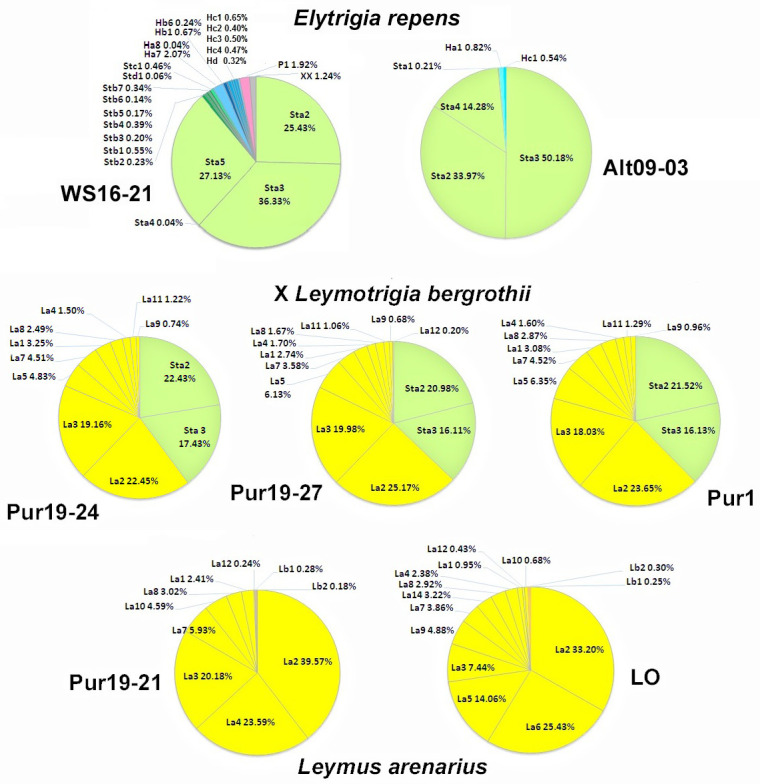
Ribotype composition diagram reflecting intragenomic polymorphism of the ITS1 sequences in ×*Leymotrigia bergrothii* and their parent species.

St-ribotypes are characteristic of *Elymus, Elytrigia,* and ×*Leymotrigia*. They are easily recognized by the presence of a characteristic 4-nucleotide insertion in ITS1 (Supplementary Table S2). H-ribotypes were found in *Hordeum* as well as a minor fraction in *Elytrigia*. We did not detect H-ribotypes in the ×*Leymotrigia* genome. In all three samples of ×*L. bergrothii*, only St- and La-ribotypes were present. Their ratio (St/La) was similar for all individuals and was approximately 40/60 (voucher Pur19-24), 37/63 (Pur19-27), and 38/62 percent (Pur1) (Figure 4 and Figure 5).

In our study, ribotypes of the P family were a characteristic feature of the *Agropyron* genomes and were found as minor fractions in *Elymus* and *Elytrigia*. *Leymus* species carry two ribotype families, major и minor, La, and Lb, correspondently. *Psathyrostachys juncea* has only Ns ribotypes (Figure 4). These ribotypes were not found in the genome of ×*Leymotrigia* (Figure 4). A correspondence was traced between the presence of ribotype families in the examined species and the type of the genome of the genus (H, St, Xm, etc.), as identified through cytogenetic and molecular phylogenetic studies [27,34,35].

**Figure 6:**
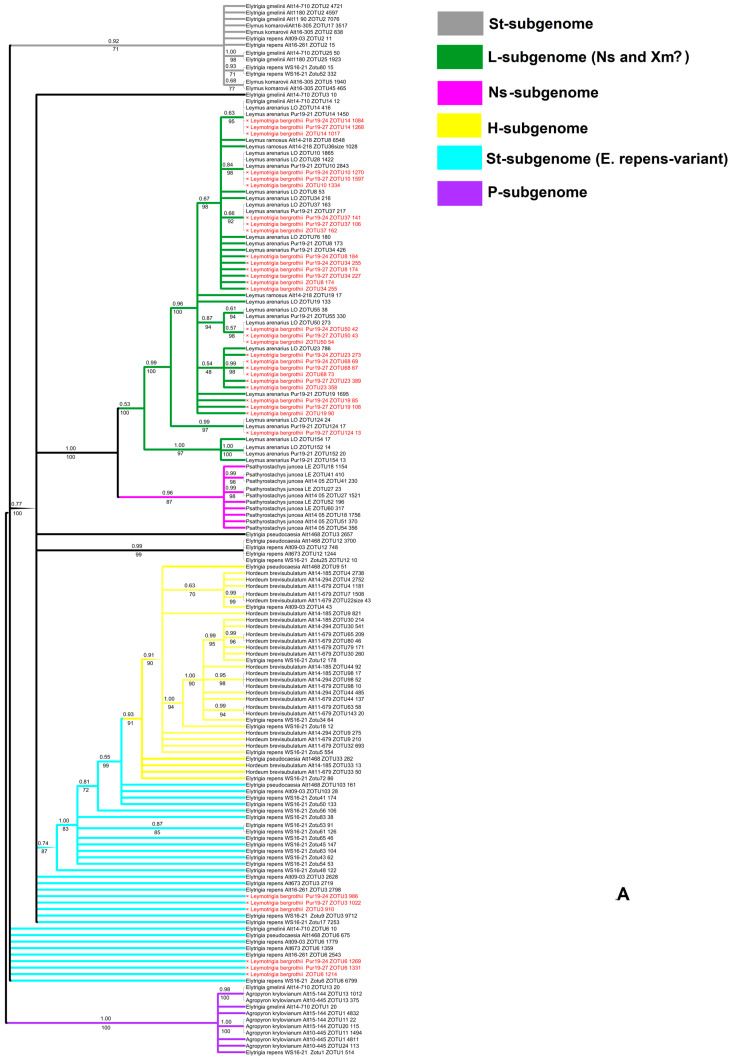

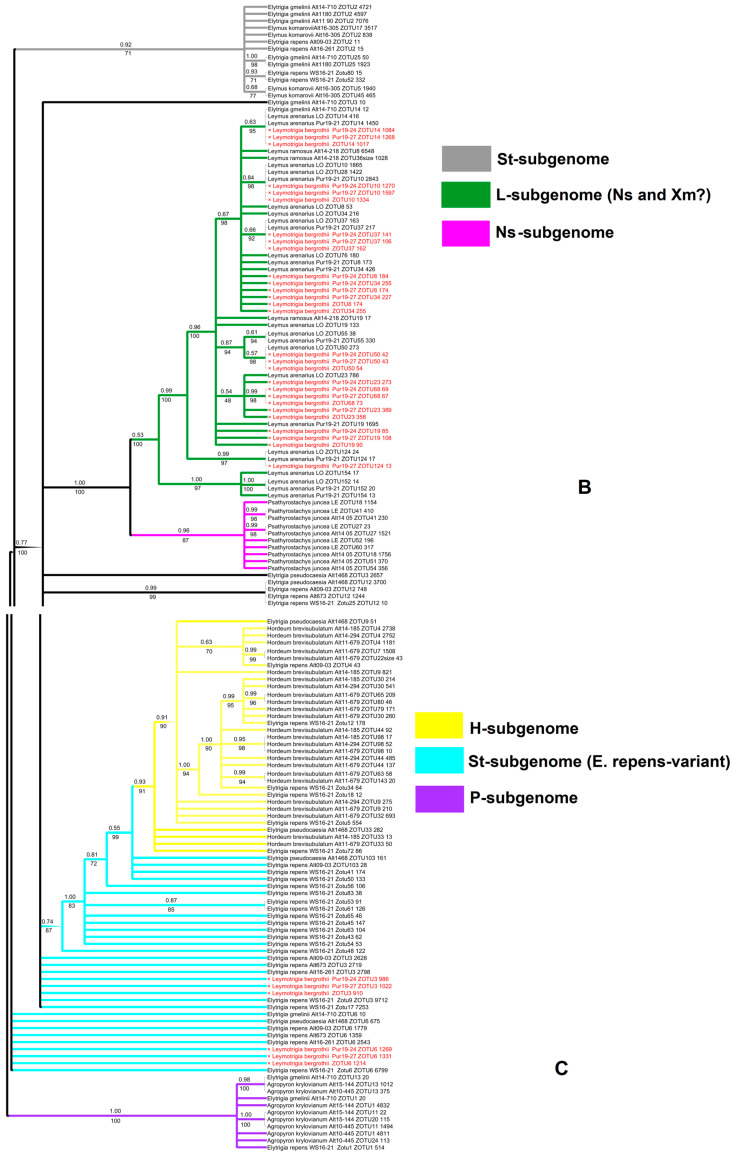
(**A**) Ribotype tree of the studied species. Index above the branch shows Bayesian support (PP); index below the branch is a bootstrap index. (**B**) A more detailed picture of the relationships between St, L, and Ns subgenomes. (**C**) A more detailed picture of the relationships between H, St (Elytrigia repens-variant), and P subgenomes.

The ribotype tree based on NGS analysis showed a presence of the subgenomes St, Ns, H, and P (Figure 6A). The ribotype tree showed that the subgenome of *Psathyrostachys* (Ns) is monophyletic with the *Leymus* subgenome (here, named L) (PP = 1, BS = 100), but they are not identical (Figure 6A,B). The Ns subgenome of *Psathyrostachys* forms moderately to a strongly supported clade (PP = 0.96, BS = 87, Figure 6A,B). As we see, the rDNA of the hybrid ×*Leymotrigia bergrothii* was inherited from both *Leymus arenarius* and *Elytrigia repens* (Figure 6A). The ribotypes of ×*L. bergorthii* that belong to *E. repens* line differed from the St-genome variant that was borrowed from the sect. *Pseudoroegneria* (*Elytrigia gmelinii*) (Figure 6B). Some St-genome variants of *E. repens* occupied an uncertain position in the clade (major ribotypes), whereas other St-ribotypes of *E. repens* were the sisters of the H subgenome (PP = 0.74, BS = 84, Figure 6A,B). Only a minor fraction of rDNA from *Elytrigia* (the ribotypes from *E. repens*, White Sea and *E. pseudocaesia* from Altai Republic) belonged to the H subgenome clade (Figure 6C).

## 3. Discussion

Interspecific hybridization, often accompanied by whole-genome duplication (WGD), is widespread in plants [36]. In the first generations of both homoploid hybrids and neopolyploids, 35S rRNA loci of only one of the parents are often transcribed [37,38]. This phenomenon is known as nucleolar dominance. However, as a rule, rDNAs from both parent species physically exist in hybrid genomes at this stage [39,40,41]. Over generation, rDNA homogenization occurs, leading to the absence or minimal representation of 35S rDNA derived from one of the ancestral species [25,42]. Rare 35S rDNA variants cannot be detected by a Sanger sequencing approach unless there is the preliminary cloning of ITS regions [19]. However, the application of next-generation sequencing (NGS) techniques allows for the detection of even minor variants of rDNA inherited from progenitors in allopolyploid genomes [19,21,22,38]. This is the approach we employed in this present study.

The study of several Triticeae species presented here has shown that the ribotype patterns of all examined species correspond well to the basic genomes (subgenomes) of the genera, as identified through cytogenetic and molecular phylogenetic studies [27,35]. In particular, the seven closely related ribotypes (ZOTUs) found in the diploid *Psathyrostachys juncea* differ from each other by only a single SNP. Obviously, all of them correspond to the rDNA variants of the Ns subgenome of *Psathyrostahys* [35,43]. Similarly, the four ribotypes found in the genomes of *Agropyron krylovianum* correspond to the P subgenome of *Agropyron* [35,43]. The ribotypes of *Hordeum brevisubulatum* are more diverse. They can be divided into two subfamilies called Ha and Hb. Specific variants of H family ribotypes, Hc and Hd, were found as a minor fraction in *Elytrigia*.

All species of the genus *Leymus* are allopolyploids with a genomic formula XmNs [35,44]. Accordingly, there are two families of ribotypes, major La and minor Lb, in the genome of *Leymus arenarius* (Figure 4). Earlier, it was shown that in the *Leymus* polyploid species examined, ITS variants of the Ns subgenome are significantly more abundant than those of the Xm subgenome (the majority of species had only the Ns subgenomic type of ITS sequences) [45]. It seems that the La ribotypes correspond to the rDNA of the *Leymus* Ns subgenome. In this case, one can assume that the Lb ribotypes are indicators of the Xm subgenome.

In our study, the ribotype P family was found not only in the genomes of *Agropyron* but also in *Elymus/Elytrigia* genomes. This is likely a result of relatively recent acts of interspecific hybridization between *Agropyron* and *Elymus*, as was previously described [27,46].

*Elytrigia repens*, like all other species of *Elymus* and *Elytrigia*, is an allopolyploid with a similar genomic composition consisting of St and H subgenomes, as was shown by studying meiotic pairing in interspecific hybrids, GISH patterns, and nuclear single-copy gene sequence information [27,47,48]. The H-ribotype sequences, characteristic of *Hordeum* subgenomes, were identified in only one plant of *Elytrigia repens* collected in the Altai Mountains and in *E. repens* from the White Sea coast (Figure 4, Table S2). In both cases, this constituted a minor fraction, not exceeding 3% of the total number of reads. This corresponds well with the molecular phylogenetic studies and FISH results. The H-family of ITS sequences was not detected by Sanger sequencing of ITS1-5.8S rDNA-ITS2 of *Elymus* and *Elytrigia* [49,50]. Furthermore, Mahelka and Kopecky [49] showed that in the *E. repens* karyotype, all rDNA loci except one minor were located on *Pseudoroegneria*-derived chromosomes, suggesting the loss of most rDNA loci derived from *Hordeum* during genome fractionation [51], similar to that which occurred with the rDNA of *Zingeria biebersteiniana* in the allotetraploid genome of *Z. trichopoda* [52].

Comparison of the chloroplast *trn*L gene sequences of *Leymus arenarius, Elytrigia repens*, and ×*L. bergrothii* showed that this chloroplast DNA region is identical in ×*Leymotrigia bergrothii* and *Elytrigia repens (*Table S2). Since it is known that in cereals, such as wheat, triticale, barley, and maize, both generative and sperm cells regularly contain plastids, but they are not transmitted into the egg cell [53,54], it means that *Elytrigia repens* was the mother plant of the hybrid nothospecies ×*Leymotrigia bergrothii*.

A study of the karyotype of ×*Leymotrigia bergrothii* showed that there are 49 chromosomes in the somatic cells of the root meristem (Figure 2). It is a homoploid, as one of the parental species, *Leymus arenarius,* has 2n = 56 [1,55,56], and there are 2n = 42 in the karyotype of maternal species *E. repens* [1,55]. The chromosome number for ×*Leymotrigia bergrothii* was identified for the first time in this species.

The genomic composition of the three studied samples of ×*Leymotrigia bergrothii* was identical. The major ribotypes of the parental species—*Elytrigia* and *Leymus*, St- and Xm—respectively, are harbored in the nuclear genome of ×*Leymotrigia bergrothii* in almost equal proportions. Therefore, if we followed the Löve and Dewey’s generic concept [26,34,57], the species would belong to the genus ×*Elyleymus* B. R. Baum.

It was unexpected that ×*Leymotrigia bergrothii*, unlike the allopolyploid species of the genus *Avena* [58] and intergeneric hybrid ×*Elyhordeum* [50], does not have a large number of rare ITS1 variants originating from the major ribotypes. The appearance of a large number of derivatives of one or several “parental” ribotypes is associated with the process of fractionation of neohomoploid and neopolyploid genomes [50]. It can be assumed that their absence in ×*Leymotrigia* is due to the predominantly vegetative reproduction of ×*Leymotrigia bergrothii*.

## 4. Materials and Methods

**Figure 7:**
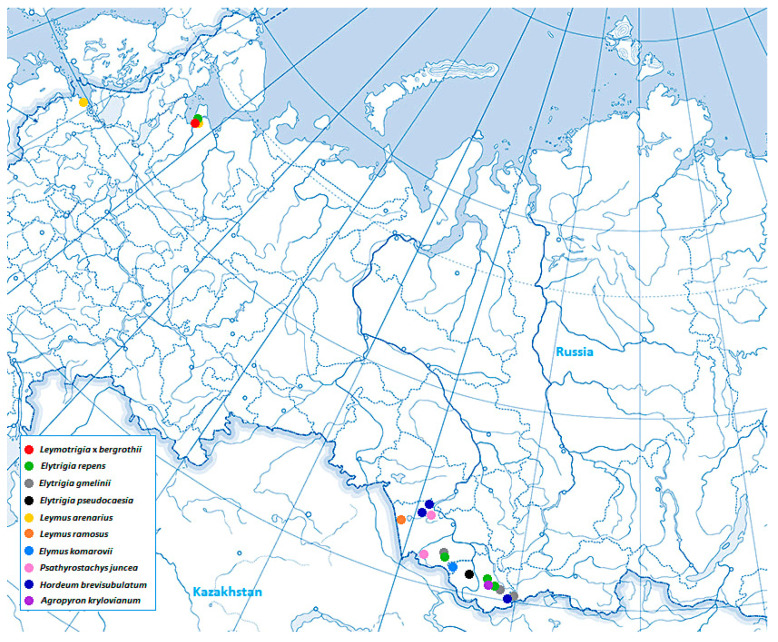
The geographic locations of sample collection.

**Figure 8:**
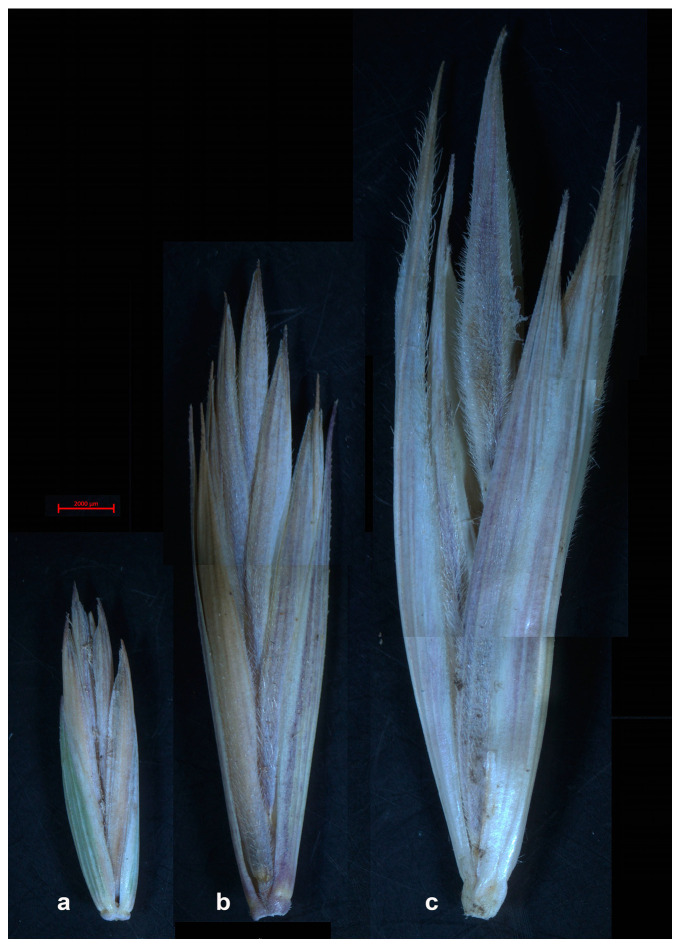
Spikelets of (**a**) *Elytigia repens* (voucher WS16-21); (**b**) × *Leymotrigia bergrothii* (voucher Pur19-27); (**c**) *L. arenarius* (voucher Pur19-21).

The herbarium specimens representing 10 species from the genera *Elymus, Leymus, Elytrigia,* ×*Leymotrigia, Hordeum, Agropyron,* and *Psathyrostachys* were collected by the authors during expeditions in Leningrad Oblast, Arkhangelsk Oblast, Altai Krai, and the Altai Republic (Russia). Plants of ×*Leymotrigia bergrothii* were collected on the eastern shores of Onega Bay in the settlement of Purnema, Archangelsk Oblast, Russia (64°22′53″ N 37°25′57″ E). In this area, this species is one of the most abundant grass species along the coastal sandy strip. In addition, one specimen of *Psathyrostachys juncea* was taken from the LE Herbarium (Komarov Botanical Institute, St. Petersburg, Russia). Plant identification was carried out according to the N.N. Tzvelev and N.S. Probatova monograph [1]. The list of herbarium specimens that were subsequently taken for sequencing is shown in Table 1. The geographic locations of the sample collection are shown in Figure 7. Figure 8 shows spikelets of *E. repens*, ×*L. bergrothii* and *L. arenarius*. It is evident that in addition to intermediate spikelet size, the hybrid has intermediate features of the glumes, lemmas, and paleas of the parent species, such as the degree of pubescence and arrangement of hairs and bristles.

To detect the hybrid status of the plants of ×*L. bergrothii* collected on the east coast of Onega Bay, we performed pollen fertility analysis as described earlier [20]. Briefly, one or two anthers were taken from the herbarium material and placed in an Eppendorf tube with 200−250 µL of 45% acetic acid for 1 h. Then, each anther was placed on a glass slide, 50 μL of 1% acetocarmine solution was added, and the anther was covered with a coverslip and slightly heated. After that, the material was flattened under the coverslip by lightly tapping with a wooden stick. Then, under a microscope, colored and uncolored pollen grains were counted. Unstained pollen grains without cytoplasmic contents or slightly colored as well as deformed ones were considered abortive, while uniformly intensely colored and undeformed ones were considered conditionally fertile.

The ploidy level of ×*L. bergrothii* was determined by direct chromosome counting on metaphase plates from the root meristem of plants collected from natural habitats and cultivated in pots, using a slightly modified aceto-orcein method [59]. The roots were washed with distilled water and treated with a 0.05% colchicine solution for 2 h. The root tips were then macerated in 45% acetic acid for 10–15 s at 60 °C. Samples were subsequently washed three to five times with distilled water to remove any residual hydrochloric acid and stained with 1% acetic carmine for 2 h, followed by squashing in 45% acetic acid. 

Total genomic DNA was isolated using the CTAB method [60] with minor modifications. The chloroplast gene *trn*L-UAA was amplified with the primers tabC and tabD [61]. Amplification parameters were as follows: one cycle of 95 °C for 5 min; 35 cycles: 95 °C for 40 s; 52–56 °C for 40 s; 72 °C for 40 s; and final elongation 72 °C for 10 min. PCR was performed in 15 μL of the reaction mixture containing 0.5–1 units of activity of Q5^®^ High-Fidelity DNA Polymerase (NEB, Ipswich, MA, USA), 5 pM of forward and reverse primers, 10 ng of DNA template, and 2 nM of each dNTP (Life Technologies, ThermoScientific, Waltham, MA, USA). The ITS1 was amplified with primers ITS 1P [62] and ITS 2 [63]. The PCR was performed under the following conditions: 94 °C for 1 min, followed by 25 cycles of 94 °C for 30 s, 55 °C for 30 s, 72 °C for 30 s, and a final elongation 72 °C for 5 min. PCR products were then purified according to the Illumina recommended method using AMPureXP (Beckman Coulter, Indianapolis, IN, USA). The libraries for sequencing were prepared according to the manufacturer’s MiSeq Reagent Kit Preparation Guide (Illumina) (http://support.illumina.com/documents/documentation/chemistry_documentation/16s/16s-metagenomic-library-prep-guide-15044223-b.pdf, accessed on 20 January 2022. They were sequenced on an Illumina MiSeq instrument (Illumina, San Diego, CA, USA) using a MiSeq^®^ ReagentKit v3 (600 cycles) with double-sided reading (2 × 300 bp) following the manufacturer’s instructions. The sequences were trimmed with Trimmomatic [64], included in Unipro Ugene [65] using the following parameters: PE reads, sliding window trimming with size 4, quality threshold 12, and minimal read length 130. Further, paired marker sequences were combined, dereplicated, and sorted into ZOTU (zero-radius operational taxonomic units) or ribotypes with the aid of vsearch 2.7.1 [66]. Only reads with 100% sequence similarity, the so-called zero-radius operational taxonomic units (ZOTUs) [67], were considered. Note that ZOTUs and ribotypes are synonymous. Singletons (unique reads that are potential sequencing artifacts) and all ITS1 sequence variants (ZOTUs) read fewer than 10 times were removed, and the remaining ITS1 reads were used for all further analyses. Ribotypes (ZOTUs) were BLASTed against the nucleotide database to filter out off-target sequences other than ITSs of grasses. Then ribotypes read 10 or more times were analyzed by TCS 1.21 [32] and visualized in tcsBU [33]. The resulting ribotype network was built according to the statistical parsimony algorithm and shows both ribotype variations and possible hybridization events in the species complex. 

## Figures and Tables

**Table 1:** List of the species included in this present study, the locations of the specimens, and GenBank numbers of the sequences.

Species	Voucher Number	GenBank Number, ITS1, NGS Data	GenBank Number, *trn*L–*trn*F	2n	Location of the Specimen
*Agropyron krylovianum* Schischk.	Alt10-445 Alt15-144	PP153840–PP153843 PP153642–PP153645		?	Altai Republic Altai Republic
*Elymus komarovii* (Nevski) Tzvelev	Alt16-305	PP153758–PP153761		28	Altai Republic
*Elytrigia gmelinii* (Trin.) Nevski (syn. *Pseudoroegneria gmelinii* (Trin. ex Schrad.) Sennikov)	Alt14-710 Alt1180 Alt11-90	PP153629–PP153635 PP153636–PP153637 PP153638		14	Altai Krai Altai Republic Altai Republic
*Elytrigia pseudocaesia* (Pacz.) Prokudin (syn. *Elymus repens* (L.) Gould subsp. *pseudocaesius* (Pacz.) Melderis)	Alt1468	PP153646–PP153651		42	Altai Republic
*Elytrigia repens* (L.) Nevski (syn. Elymus repens (L.) Gould)	Alt09-03 Alt673 Alt16-261 WS16-21	PP153765–PP153770 PP153771–PP153773 PP153774–PP153776 PP153859–PP153888	PQ318204 PQ318205 PQ318206	42	Altai Republic Altai Republic Altai Republic Arkhangelsk Oblast
*Hordeum brevisubulatum* (Trin.) Link	Alt14-185 Alt14-294 Alt11-679	PP153658–PP153663 PP153664–PP153668 PP153669–PP153682		28	Altai Krai Altai Krai Altai Republic
*×Leymotrigia bergrothii* (H. Lindb.) Tzvelev (syn. *×Elyleymus bergrothii* (H.Lindb.) Conert)	Lt Pur19-24 Pur19-27	PP153830–PP153839 PP153806–PP153816 PP153817–PP153828	PQ318211 PQ318209 PQ318210	49	Arkhangelsk Oblast Arkhangelsk Oblast Arkhangelsk Oblast
*Leymus arenarius* (L.) Hochst.	LO Pur19-21	PP153782–PP153795 PP153796–PP153805	PQ318207 PQ318208	56	Leningrad Oblast Arkhangelsk Oblast
*Leymus ramosus* (K.Richt.) Tzvelev	Alt14-218				Altai Krai
*Psathyrostachys juncea* (Fisch.) Nevski	LE4 Alt14-05	PP153732–PP153737 PP153738–PP153742		14	Altai Krai, LE Altai Krai

## Data Availability

Data are contained within the article or Supplementary Materials.

## Supplementary Materials

The following supporting information can be downloaded at: https://www.mdpi.com/article/10.3390/ijms252211966/s1.
